# *Haemophilus pittmaniae* respiratory infection in a patient with siderosis: a case report

**DOI:** 10.1186/1752-1947-6-120

**Published:** 2012-04-30

**Authors:** Mathilde Bouc Boucher, Marielle Bedotto, Carine Couderc, Carine Gomez, Martine Reynaud-Gaubert, Michel Drancourt

**Affiliations:** 1 Laboratoire de Microbiologie, Institut Hospitalier Universitaire POLMIT, Assistance Publique-Hopitaux de Marseille, Marseille, France; 2 Service de Pneumologie, Équipe de Transplantation Pulmonaire, Assistance Publique-Hopitaux de Marseille, CHU Nord, Faculté de Médecine, URMITE CNRS-UMR 6236, Aix-Marseille Université, Marseille, France

## Abstract

**Introduction:**

*Haemophilus pittmaniae* was described in 2005 as a new species distantly related to *Haemophilus parainfluenzae*. This member of the human saliva microbiota has also been further isolated from various body fluids without formal description of the patients.

**Case presentation:**

We report the case of *H. pittmaniae* isolate made from a sputum specimen collected from a 58-year-old Caucasian man with a massive fibrotic form of siderosis who was awaiting lung transplantation. Identification of the isolate was ascertained by matrix-assisted laser desorption/ionization time-of-flight mass spectrometry and 16S rRNA gene sequencing. *H. pittmaniae* was considered to be responsible for the worsening of the patient’s chronic respiratory failure and was successfully treated with oral amoxicillin.

**Conclusion:**

*H. pittmaniae* should be regarded as a new pathogen responsible for respiratory tract infection in patients with chronic lung diseases.

## Introduction

*Haemophilus pittmaniae* has recently been delineated as a new member within the genus *Haemophilus* after multilocus sequence analysis of nine human isolates collected from six different clinical sources [1]. This organism has been determined as being part of the normal oral microbiota of humans [1]. Phylogenetic and phenotypic studies have shown that *H. pittmaniae* belongs to the phylum Proteobacteria within the family *PasteurellaceaeHaemophilus parainfluenzae* cluster. In our present report on *H. pittmaniae*, we found no previous clinical description of patients with this entity, hence hampering the interpretation of isolation of this organism in clinical specimens. We report the case of a patient which provides evidence for *H. pittmaniae* as an organism involved in respiratory tract infection.

## Case presentation

A 58-year-old Caucasian man was admitted to the Department of Pulmonary Disease at our institution for worsened chronic respiratory failure. He had an end-stage fibrotic form of siderosis and had been evaluated 3 months earlier as candidate for lung transplantation. At admission, the patient presented with fever at 38.5°C, cough with mild productive sputum, an important increased dyspnea on exertion and severe hypoxemia requiring oxygen supplementation of 12L/minute at rest to obtain a systemic oxygen saturation of 94%, whereas he had required 6L/minute at rest 3 months before this presentation. His peripheral white blood cell count was 15.2 g/L, and neutrophils were 10.6g/L. There was no other organ dysfunction. A chest X-ray (CXR) and computed tomographic (CT) scan were obtained on admission. The radiological findings confirmed a pattern of advanced fibrosis. There were no significant changes or new findings compared to the CXR and CT scan obtained 3 months previously.

He had stopped smoking 5 months before his current presentation, and his body mass index was 29.4kg/m^2^. His usual treatment consisted of nebulized terbutaline 5mg and ipratropium bromide 0.5mg, three times daily, together with oral prednisone 40mg/day tapered to 30mg/day during this hospitalization. In this context, an expectorated sputum specimen was collected for microbiological investigations. *H. pittmaniae* was isolated in this specimen. Other causes of respiratory decompensation were excluded, and his respiratory virus panel and fungi were negative. Following the detection of *H. pittmaniae*, a single antibiotic, oral amoxicillin 3g/day, was administered for 10 days, which had a rapid clinical benefit, permitting his oxygen supplementation to be reduced to the previous dosage of 6L/minute at rest and allowing him to await the single-lung transplantation, which was performed successfully 2 months later.

Direct microscopic examination of the sputum specimen showed the presence of numerous polymorphonuclear leukocytes and a few epithelial cells, but no bacteria were detected after Gram staining. The sputum specimen was cultured on chocolate agar (BioMérieux, Marcy l’Etoile, France), incubated under a 5% CO_2_-enriched atmosphere at 37°C and on Columbia agar (BioMérieux) under aerobic and anaerobic atmospheres at 37°C. Antibiotic susceptibility testing was performed by determining the minimum inhibitory concentration to amoxicillin, amoxicillin-clavulanate, ceftriaxone, gentamicin, ciprofloxacin, erythromycin and doxycycline using the disk diffusion method on a chocolate agar (BioMérieux) incubated under a 5% CO_2_-enriched atmosphere at 37°C and read after 48-hour incubation. The presence of β-lactamase was tested by using the cefinase test with a paper disk impregnated with nitrocefin (Becton Dickinson, Le Pont de Claix, France) in the presence of a β-lactamase-producing *H. influenzae* isolate as a positive control.

The chocolate agar showed grayish white colonies after 48-hour incubation in aerobic atmosphere, whereas the Columbia agar plates remained sterile after 5-day incubation in both aerobic and anaerobic atmospheres. Gram staining revealed Gram-negative bacilli susceptible to β-lactam and ciprofloxacin (Table 1). The cefinase test was negative.

**Table 1:** **Antibiotic susceptibility pattern of a** ***Haemophilus pittmaniae*** **isolate from sputum**

**Antibiotic**	**Minimum inhibitory concentration (mg/ml)**
Amoxicillin	<1
Amoxicillin + clavulanic acid	2
Ceftriaxone	<0.5
Gentamicin	>2
Ciprofloxacin	0.5
Erythromycin	>8
Doxycycline	8

**Figure 1:**
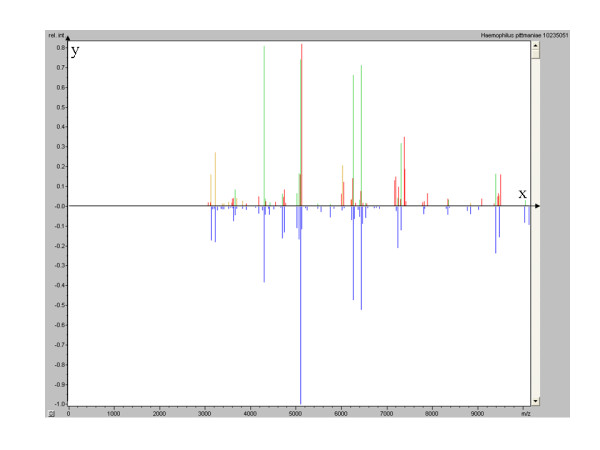
**Brüker Daltonics flexAnalysis of*H. pittmaniae*(upper panel) and*H. parainfluenzae*(lower panel) MALDI-TOF mass spectrometry protein spectra.** X axis, m/z value of peaks; y axis, relative intensity of peaks.

One colony was harvested and deposited on MTP 384 target plate polished steel (Brüker Daltonik GmbH, Bremen, Germany) in four replicates. Two microliters of matrix solution (saturated α-cyano-4-hydroxycinnamic acid, 50% acetonitrile and 2.5% trifluoroacetic acid) were then added, and samples were air-dried for 5 minutes before being processed in the mass spectrometer. Measurements were performed using an autoflex II matrix-assisted laser desorption/ionization time-of-flight (MALDI-TOF) mass spectrometer (Brüker Daltonik GmbH). Four raw spectra were automatically acquired using Brüker Daltonik’s flexControl version 3.0 software and then compared with the Brüker Daltonik database and MALDI Biotyper version 2.0 software (Brüker Daltonik GmbH). To validate the analysis of a whole MTP 384 target, bacterial test standard (protein extract of *Escherichia coli* DH5α, 255343; Brüker Daltonik GmbH) and matrix solution were added to the analysis batch as positive and negative controls, respectively. Two deposits of 1.5 μl of bacterial test standard covered with 1.5 μl of matrix were used to calibrate the Brüker Daltonik Biotyper analysis and as a validation standard for the analysis. To validate the analysis using the Maldi Biotyper software, the bacterial identification must be *E. coli* with an identification score >2 and the two negative controls’ deposits of 1.5 μl of matrix solution must give a score <1.7. In MALDI-TOF mass spectrometric analysis, negative controls yielded faint spectra with non-significant identification scores and the *E. coli* positive control yielded the expected spectrum. The isolate yielded four spectra (Figure 1), and the comparison with the Brüker Daltonik database yielded identification of *H. pittmaniae* three times, with scores of 2.085, 2.062 and 2.081. The fourth colony was identified as *H. parainfluenzae*, with a score of 2.014.

Bacterial DNA was extracted by using a MagNA Pure LC DNA Isolation Kit III (Roche Diagnostics, Meylan, France) with a MagNA Pure LC instrument according to the manufacturer’s instructions. Polymerase chain reaction (PCR) amplification of the 16S rRNA gene was performed by using the universal primer pair fD1 (5′-AGAGTTTGATCCTGGCTCAG-3′) and rP2 (5′-ACGGCTACCTTGTTACGACTT-3′) [2,3] in an ABI 2720 thermal cycler (Applied Biosystems, Courtaboeuf, France). PCR mix without DNA was used as a negative control. PCR products were purified by using a MultiScreen PCR filter plate (Millipore, Molsheim, France), and sequencing reactions were carried out by using a DNA sequencing kit (BigDye Terminator Cycle Sequencing Ready Reaction Kit version 1.1; Applied Biosystems) according to the manufacturer’s instructions. Sequencing products were purified and electrophoresis was performed with a 3130 Genetic Analyzer (Applied Biosystems). Sequences obtained were analyzed with AutoAssembler software (Applied Biosystems) and Sequencher software (Gene Codes Corp, Ann Arbor, MI, USA) and compared with those available in the GenBank database by using the BLAST program (http://blast.ncbi.nlm.nih.gov/Blast.cgi). PCR yielded a unique product, but negative controls remained negative. The 1464-bp 16S rRNA gene sequence was compared with sequences deposited in the GenBank database and was found to be 99% similar to that of *H. pittmaniae* (NR 025423.1, strain HK85). The next closest bacteria was *H. parainfluenzae* with a 97% similarity score.

## Discussion

In our present case report, we describe the first *H. pittmaniae* isolate found in our laboratory, suggesting that this isolate did not result merely from contamination but was indeed present in the sputum specimen, despite the fact that it was not detected by direct microscopic examination. The isolate was taken from a specimen collected from a patient at the time of worsening chronic respiratory failure. Accordingly, the patient’s condition rapidly improved when he was started on amoxicillin, an antibiotic therapy guided by the *in vitro* susceptibility pattern of the *H. pittmaniae* isolate. These data suggest that this *H. pittmaniae* strain may have played a role in our patient’s worsening respiratory function, along with a potential facilitating role of infection by maintenance therapy with steroids. Indeed, the only previous report of this new organism lacked any clinical detail that would have allowed the interpretation of this rarely encountered bacterium. Nevertheless, that initial description of *H. pittmaniae* reported this new species as an opportunistic pathogen isolated from blood and bile in humans. The data reported herein suggest the respiratory tract as an additional site of opportunistic *H. pittmaniae* infection [3].

In this case report, the accurate identification of the isolate was ensured by both MALDI-TOF mass spectrometry and 16S RNA gene sequencing after *H. pittmaniae* was identified on the basis of multilocus sequence analysis [1]. Whereas this labor- and time-intensive method cannot be used for the routine identification of *Haemophilus* spp. isolates, MALDI-TOF mass spectrometry proved to be the most rapid and cost-effective tool for identification, as previously reported for bacteria in general [5]. We previously observed that accurate MALDI-TOF mass spectrometry-based identification of bacteria required a minimum of 10 reference spectra in the database [5]. In our present case, despite the fact that the Bruker Daltonik database included only one *H. pittmaniae* reference spectrum, we obtained three of four spectra corresponding to this identification, whereas one of the four spectra gave a *H. parainfluenzae* identification with a lower score. These two bacteria have close, albeit distinct, spectra (Figure 1), which is in agreement with their taxonomic relationships as assessed by 96.7% similarity in the *sod*A sequence [5]. 16S rRNA gene sequence analysis previously revealed that both *H. pittmaniae* and *H. parainfluenzae* were not related to the “*Haemophilus sensu stricto*” group [1,4].

## Conclusion

*H. pittmaniae* is a rare opportunistic pathogen which can worsen chronic respiratory failure. MALDI-TOF mass spectrometry is a first-line method for identification of *H. pittmaniae* isolates. This case report helps in the interpretation of *H. pittmaniae* as an opportunistic pathogen requiring appropriate antibiotic treatment to improve the patient’s health status.

## Consent

Written informed consent was obtained from the patient for publication of this case report and any accompanying images. A copy of the written consent is available for review by the Editor-in-Chief of this journal.

## Competing interests

The authors declare that they have no conflict of interest regarding this work.

## Authors’ contributions

MBB, MRG and MD reviewed the clinical data and were major contributors to the writing of the manuscript. MRG was involved with patient management. MBB, BM and CC analyzed the data and performed the laboratory analysis. All authors read and approved the final manuscript.
